# Humans adjust their grip force when passing an object according to the observed speed of the partner’s reaching out movement

**DOI:** 10.1007/s00221-018-5381-5

**Published:** 2018-09-27

**Authors:** Marco Controzzi, Harmeet Singh, Francesca Cini, Torquato Cecchini, Alan Wing, Christian Cipriani

**Affiliations:** 1 The BioRobotics Institute, Scuola Superiore Sant’Anna, Pisa, Italy; 2 School of Psychology, University of Birmingham, Birmingham, UK

**Keywords:** Object handover, Passing, Grip force, Predictive control, Visual information, Human-robot collaboration

## Abstract

The way an object is released by the passer to a partner is fundamental for the success of the handover and for the experienced fluency and quality of the interaction. Nonetheless, although its apparent simplicity, object handover involves a complex combination of predictive and reactive control mechanisms that were not fully investigated so far. Here, we show that passers use visual-feedback based anticipatory control to trigger the beginning of the release, to launch the appropriate motor program, and adapt such predictions to different speeds of the receiver’s reaching out movements. In particular, the passer starts releasing the object in synchrony with the collision with the receiver, regardless of the receiver’s speed, but the passer’s speed of grip force release is correlated with receiver speed. When visual feedback is removed, the beginning of the passer’s release is delayed proportionally with the receiver’s reaching out speed; however, the correlation between the passer’s peak rate of change of grip force is maintained. In a second study with 11 participants receiving an object from a robotic hand programmed to release following stereotypical biomimetic profiles, we found that handovers are experienced as more fluent when they exhibit more reactive release behaviours, shorter release durations, and shorter handover durations. The outcomes from the two studies contribute understanding of the roles of sensory input in the strategy that empower humans to perform smooth and safe handovers, and they suggest methods for programming controllers that would enable artificial hands to hand over objects with humans in an easy, natural and efficient way.

## Introduction

Object handover between a passer and a receiver is probably one of the most basic collaborative motor tasks between humans. Handover is fundamental for a wide range of functional and social activities in which individuals help each other, sharing the same goal and a common plan of execution. Handover may be divided in three phases (Cutkosky and Hyde 1993; MacKenzie and Iberall 1994; Mason and MacKenzie 2005). First, the passer and the receiver coordinate their movements, through non-verbal communication, to reach the exchange site, at a given time. The end of this phase is marked by a mild collision between the partners when the receiver makes contact with the object. Second, while the receiver increases grip force (GF) on the object the passer decreases it. During this time, both partners coordinate the modulation of GF to counteract gravitational and inertial load forces to prevent the object from slipping. Third, once the receiver stably holds the object, the handover action is completed and the passer removes his or her hand from the exchange site.

While simple prehensile movements such as reaching to grasp have been widely explored during both individual or joint actions (Castiello 2005; Georgiou et al. 2007; Becchio et al. 2008a, b), to date few investigators have studied the way GFs are modulated by two partners to handover an object. Unlike reaching to grasp, where feedforward and feedback mechanisms reside within a single control system (Jeannerod 2003), handover involves multiple, disconnected control systems (Mason and MacKenzie 2005). Thus, an important theoretical question is how the disconnected neural controllers are coordinated to produce fluent handover? A second important question is what are the relative contributions of feedforward vs. feedback mechanisms in the control of handover? Continuous closed-loop control of dynamic motor behaviours is severely limited by neural delays, and therefore, impractical at frequencies above 1 Hz (Hogan et al. 1987). Hence, continuous modulation of GF based only on incoming tactile/haptic information (i.e. a purely feedback mechanism) cannot explain the coordination of rapid and stereotypical movements, such as those in handover. Predictive mechanisms combined with a neural system that mimics the motor control and the behaviour of objects in the external environment (internal models) (Kawato and Wolpert 1998), must also be involved. Nevertheless, some feedback control is likely to be necessary given the actions of one partner cannot be fully predicted by the other (Mason and MacKenzie 2005).

One of the motor control policies described in the literature, posits that motor programs of tasks, such as object manipulation, are organized in phases delimited by means of multi-modally encoded discrete sensory events, e.g. resulting from object contact, lift-off, etc. (Johansson and Edin 1993; Randall Flanagan et al. 2006; Johansson and Flanagan 2009; Fang et al. 2015). The nervous system predicts such events to launch in advance the appropriate motor commands, and monitors progress to initiate, if necessary, corrective actions that are suitable for the task and the current phase. Normally the task evolves in an open-loop fashion where the successful completion of each phase is signified by specific combinations of temporally correlated sensory signals. The prediction of the sensory events is used by the brain to proceed to the next phase without delays. This control policy may apply also for the handover, the predicted collision between the receiver and the object, held by the passer, being one of the key sensory events of the task.

Mason and MacKenzie (2005) suggested that passers use visual-feedback based anticipatory control to precisely trigger the handover and provided preliminary evidence that the receiver’s reach towards the passer is used by the passer to update prediction. Quesque et al. (2016) showed that when two subjects manipulate an object in turn, the kinematics of the reaching to grasp movement of one partner may affect the motion of the arm of the other partner in the subsequent phase of the task. Furthermore, behaviour that adapts to speed has been demonstrated in other manipulative tasks such as collision (Turrell et al. 1999) and catching (Savelsbergh et al. 1992) tasks, however, to date it has not been formally assessed in handover actions. In particular, it is unclear whether the passer uses information about the dynamics of the receiver’s reaching movement to anticipate and launch the appropriate release motor program. To address this general issue, we ask: (1) does a rapidly approaching receiver trigger faster handovers by the passer? (2) is a faster handover characterized by an earlier beginning of GF release or by a more rapid reduction in GF by the passer?

Previous works (Avenanti et al. 2013; Sciutti and Sandini 2017) provided evidences that action observation as well as the knowledge of the task and context in which the action takes place support the understanding and the anticipation of the other’s movement, thus enabling the actual collaboration between agents. Therefore, prediction of the receiver’s behaviour by the passer is likely to depend on the visually-derived perception of the other’s action (how the passer expects the receiver will behave, within the current context) (Rizzolatti and Craighero 2004) or memory of the outcome of previous handover actions. Studies of lifting and repositioning tasks show that, when visual input is obstructed, humans switch from a predictive to a purely feedback control, exhibiting immature GF profiles (Kawai et al. 2001). In such cases, the GF to lift the object is primarily based on the somatosensory information acquired from the *initial contact* with the object (Johansson and Westling 1984), whereas coarse online adjustments are triggered in reaction to perceived perturbations of the load force (Cole and Abbs 1988). Similar mechanisms might explain the behaviour of a passer, during the handover, if he/she cannot see the partner’s movements. Moreover, as observed in the blindfolded pick and lift, the magnitude of the collision with the receiver might be expected to affect modulation of the passer’s GF.

In the first of the two experiments (Exp. 1) we sought to address for the first time these issues by investigating how changes in dynamics of the receiver’s reaching movement would affect GF release by the passer, who kept his or her hand in fixed position during handover. We further explored the role of visual feedback for the passer by including normal and absent vision conditions. Our setup measured the grasp forces involved during the handover and the receiver’s acceleration profiles. We expected that passers would integrate visual input about the receiver’s dynamics into their sensorimotor control to predict the onset of the handover and to modulate their release dynamics accordingly.

In a second experiment (Exp. 2), we aimed to investigate the effects of such release dynamics on the fluency of the handover experienced by the receiver. Several studies involving robotic agents investigated humans’ preferences and how their perception of fluency can be influenced during the handover task. The majority of these studies focused on the effects of the trajectory (Basili et al. 2009; Prada et al. 2013), and of the velocity profile (Huber et al. 2008a, b) of the reaching movement; on the effects of the position of both the passer’s (Cakmak et al. 2011; Strabala et al. 2013; Huber et al. 2013; Parastegari et al. 2017) and the receiver’s arm (Pan et al. 2018a, b) used to handover the object; on the effects of other subtle non-verbal clues used to initiate an handover (Strabala et al. 2012; Moon et al. 2014). Only few studies focused on the modulation of the GF during the object release. Parastegari et al. implemented a fail-safe release controller able to detect slippage events (Parastegari et al. 2016) and assessed the effects of the minimum pulling force required to collect the object from the robot on the perceived smoothness of the handover (Parastegari et al. 2018). Chan et al. (2013), using a feedback-based release controller, showed that handovers that are completed as soon as the receiver counterbalances the object weight are not perceived safe or coordinated by humans. However, neither of the previous studies investigated the effects of the release reactivity and of the handover duration on the fluency and quality of the handover perceived by the receiver.

To address this open issue, in Exp. 2, the passer was replaced by a robotic hand programmed to release the object following a stereotypical biomimetic GF trajectory (Endo et al. 2012). In this way it was possible to manipulate the onset and the duration of the object release, thus simulating different behaviours of the passer. Because of the tight temporal coordination between the passer and the receiver in a smooth handover (Mason and MacKenzie 2005), we expected that the participants would perceive handovers that are shorter or longer than average as unnatural.

Our results from Exp. 1 show that with visual input, passers clearly predict the nature and timing of the exchange adapting their onset and rate of grip force release to the contrasting receiver dynamics in the different conditions. When blindfolded the release is correlated with haptic input arising from the collision. In Exp. 2, handovers were deemed more fluent with more reactive release behaviours, shorter release durations, and shorter handover durations. Interestingly the handover was rarely experienced as too fast. These outcomes are interesting for neuroscientists and roboticists. They contribute understanding of the roles of sensory input in the strategy that empowers humans to perform smooth and safe handovers, and they may suggest ways to program robots working and interacting with humans.

## Materials and methods

### Participants

Fourteen healthy participants (5 females, all right-handed) aged 26 ± 11 years old (mean ± standard deviation) took part in the first experiment. Eleven different participants (all male; all right-handed; age 29 ± 4 years old) took part in the second experiment. None of them reported any history of somatosensory or motor impairments and all claimed to have normal or corrected vision. Informed consent in accordance with the Declaration of Helsinki was obtained from each participant before conducting the experiments. This study was approved by the local ethical committee of the Scuola Superiore Sant’Anna, Pisa, Italy. The methods were carried out in accordance with the approved guidelines.

### Experiment 1

#### Experimental set-up

**Fig. 1:**
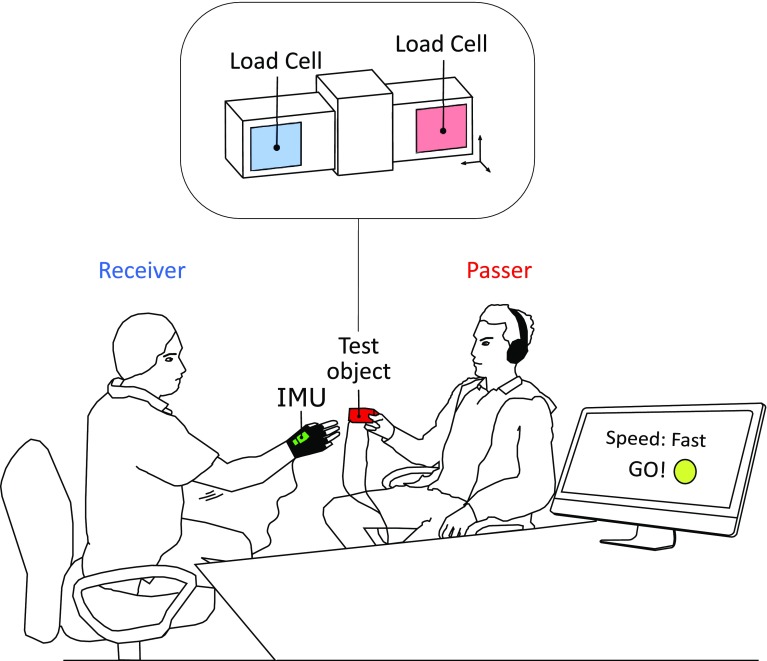
Experimental setup and test object. Subjects were seated opposite one another on two chairs. The passer was stationary while the receiver moved his upper limb toward the exchange site. A screen informed the receiver about the handover timing setup and it could not be seen by the passer. An IMU was fastened to the receiver’s moving hand. The detailed view shows the test-object instrumented with two load cells

The experimental setup consisted of a test-object instrumented to measure grasp and interaction forces and acceleration, a three-axis inertial measurement unit IMU that measured the acceleration and orientation of the receiver’s hand, and a PC (Fig. 1).

**Fig. 2:**
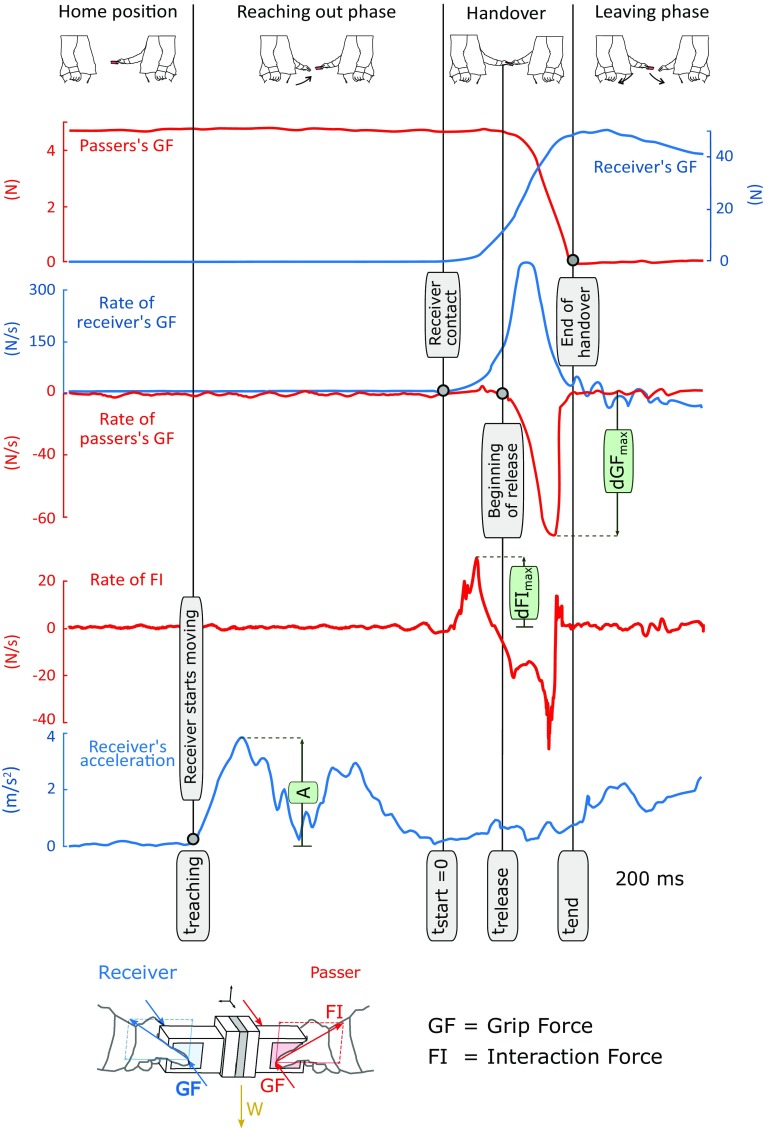
On the top is shown an example of the signals and the metric analysed in this experiment. On the bottom the force diagram of the handover is displayed

The test-object was a 3D printed symmetric plastic structure (dimensions 85 × 50 × 25 mm; weight 160 g) equipped with two six-axis force/torque (FT) sensors (model Nano17, ATI Inc., USA), similar to the object used in (Endo et al. 2012). The FT sensors recorded the passer’s and receiver’s grip force, GF, defined as the force perpendicular to the grasp surfaces, developed by the thumb opposing the index and middle finger, and the interaction force, FI, defined as the resultant force obtained by combining the horizontal force and the vertical force, of the passer and the receiver (Fig. 2). The IMU (model *3-Space Sensor*, YEI Technology, USA) tracked the receiver’s hand motion. The PC ran a custom application that acquired the data (1 kHz, NI-USB 6211, National Instruments, USA), stored it for off-line analysis, and guided the experimental protocol, in particular, by providing visual cues to the receiver (i.e. the experimenter) on the timing of the test trial.

#### Experimental protocol

In this test, the participants played the role of the passer in human–human handovers. The passer was seated comfortably on a chair wearing noise-blocking headphones and instructed to repeatedly “pass” the test-object to the receiver, sitting in front of him/her at a distance of ~ 100 cm (Fig. 1). The exchange site was at a distance of ~ 40 cm from the passer. To “pass” the object, the subject was instructed to maintain, for a maximum time of 5 s, a stationary arm posture (which was the same throughout the experiment), allowing the experimenter (the receiver) to reach towards it and take the object from his/her (the passer’s) grasp. Between the trials the passer was asked to keep the arm on the arm rest of the chair, to avoid any side effects of fatigue. Both the passer and the receiver were also instructed to use only their thumb, index and middle fingers to grasp the object (tridigital grasp) throughout the experiment. The receiver, which was the same person throughout the tests (author H.S., male, age 28), was trained to reach for the exchange site at three fixed speeds (*slow, medium*, and *fast*) and did so based on the instructions displayed on the PC screen. Notably, the passer was not informed in advance about the handover speed. Once the speed indication was displayed (at t*) to the receiver, after a random time between 1000 and 3000 ms (uniformly distributed), the receiver was prompted to begin the reaching out movement (a “go” signal). In the meanwhile, at t*, an acoustic signal was played in the passer’s headphones to alert him/her that within the next 5 s the receiver would start the handover. This procedure reduced the opportunity for the passer to anticipate the onset of the receiver’s reaching movement. For the same reason (i.e. to reduce predictability) the reaching speed of the receiver was randomly selected before each trial began. The acceleration recorded by the IMU was used to validate the trial. Trials having an acceleration modulus in the ranges 0–0.2 G, 0.2–0.5 G and 0.5–0.7 G were considered acceptable for the slow, medium and fast movements, respectively. Trials outside these ranges were automatically deleted and repeated later. The duration of the receiver’s reaching movement fell within a range of about 0.6–2 s (based on the acceleration level).

The protocol included two conditions. In one condition, the passer viewed the scene normally, and thus could use visual and tactile feedback to plan and execute the motor task (condition VT). In the other condition, the passer was blindfolded, and as such could not see the receiver’s reaching movement and had to rely on tactile feedback only (condition *T*). Each condition included 30 trials, 10 at each speed. To prevent biases in the outcomes due to learning effects, half of the passers started the experiment under condition *T*, while the other half started under condition VT. In all cases a 5–10-min break was taken between the conditions.

#### Data analysis

##### Analysis

All data were digitized and stored for off-line analysis. For each trial we identified the following relevant events of the handover: the receiver’s movement onset (*t*_reaching_), the beginning of the handover (*t*_start_), the passer’s release onset (*t*_release_), and the end of the handover (*t*_end_). *t*_reaching_ was identified using a threshold of 0.1 G on the acceleration of the receiver’s hand movement (Fig. 2). *t*_start_, defined as the instant at which the receiver made contact with the object, was identified by finding the peak rate of the receiver GF and moving backward in the trial to the first instant where it dropped below 0.08 N/s, (Fig. 2). Similarly, *t*_release_, defined as the instant at which the passer began decreasing the GF, was determined going backward from the peak rate of the GF to the first frame when the passer’s force rate exceeded − 1 N/s (Fig. 2). Notably, *t*_release_, described the passer’s reactivity (or reaction time). The procedure for identifying *t*_start_ and *t*_release_ was the one suggested by Mason and MacKenzie (2005), and prevented noise spikes in the signals from affecting segmentation of the data. Finally, the end of the handover, *t*_end_, defined as the instant when the passer broke contact with the object, was identified as the first instant when the passer’s GF fell below 5% of its maximum value (Fig. 2). The time series were synchronized on *t*_start_, i.e. when the receiver made contact with the object.

Once segmented, we also identified the following kinetic features specific to each phase of the handover: the peak absolute acceleration of the receiver during the reaching out movement (*A*), the absolute value of the peak rate of the passer’s GF during the handover (dGF_max_), and the peak rate of the interaction force (dFI_max_) before dGF_max_. Specifically dGF_max_ quantified the passer’s maximum releasing GF rate, whereas dFI_max_ the magnitude of the collision. For each subject, three Pearson’s correlation coefficients (*r*_0_, *r*_1_, *r*_2_) were computed. *r*_0_ was calculated between A and dFI_max_, for both *T* and VT conditions, to verify the existence of a relationship between the receiver’s reaching out speed and the magnitude of the haptic input. *r*_1_ was calculated between *A* and dGF_max_ under VT, while *r*_2_ was evaluated between dFI_max_ and dGF_max_ under *T*, to assess the influence of the sensory input (seen and/or perceived) on the modulation of the passer’s GF. The average of the correlation coefficients across participants was calculated performing a Fisher transformation of *r* values of each subject and back-transforming the average of the resulting *z* scores.

##### Statistical analysis

This study was characterized by two within-subjects factors, namely sensory input condition (VT and *T*) and receiver reaching speed (slow, medium, and fast). Hence a two way repeated measures ANOVA was used to assess the effects of the experimental conditions on the passer’s reactivity (*t*_release_), on the duration of the handover (*t*_end_) and on its kinetics (dGF_max_). The ANOVAs were followed by Bonferroni corrected post-hoc tests. In particular, when both the sensory input and the reaching out speed proved statistically significant a total of nine comparisons were performed; between VT and *T* for each of the reaching out speeds (three comparisons) and among *slow, medium*, and *fast* for each sensory input condition (six comparisons).

In all cases a *p* value < 0.05 was considered statistically significant. The nomenclature of the metrics used in this experiment is summarized in Table 1.

**Table 1:** Descriptive nomenclature

Symbol	Description
*A*	The peak acceleration of the receiver
dFI_max_	The peak rate of the passer interaction force
dGF_max_	The peak rate of the passer grasp force
*t*_release_	The time difference between the receiver’s object contact and the time when the passer began releasing
*t*_end_	The completion time of the transfer
*r*_0_	Average Pearson’s correlation coefficient between dFI_max_ and *A*
*r*_1_	Average Pearson’s correlation coefficient between dGF_max_ and *A*
*r*_2_	Average Pearson’s correlation coefficient between dGF_max_ and dFI_max_

### Experiment 2

#### Experimental set-up

**Fig. 3:**
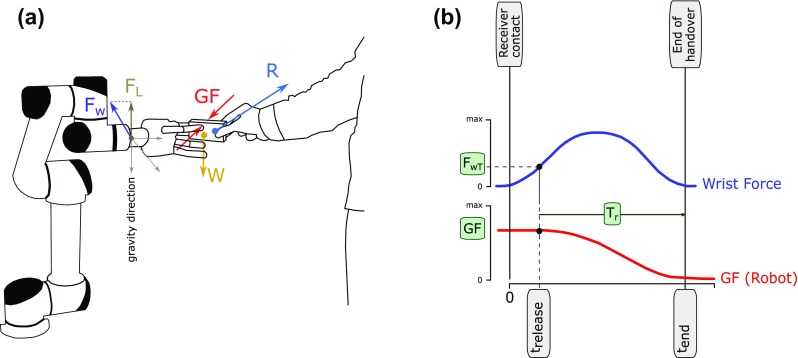
Experimental setup. **a** The robotic hand was placed in front of the receiver at a fixed height and oriented to present the test-object in a comfortable way. Force diagram of the handover. *F*_w_ is the sum of the weight of the object, *W*, with the interaction force of the receiver, *R*. The test-object is the same as used in Experiment 1. **b** Representative grip force release profile of the automatic controller used in this experiment. Release conditions differ in the threshold on the wrist force *F*_wT_ used to trigger the beginning of the handover that determines the time *T*_t_ or in the time release *T*_r_

The experimental set-up consisted of an anthropomorphic robotic hand, a three-axis force sensor, a host PC equipped with a data acquisition board and a test-object instrumented with sensors. The robotic hand was a left-handed version of the IH2 Azzurra robot hand (Prensilia Srl, Italy). Movements were limited to only flexion–extension of the thumb, index finger and middle fingers to allow stable pinch grasps between the three digits. The robotic hand included force sensors and encoders able to measure the GF and the grip aperture, respectively. The hand could implement force/position control by receiving commands from the host PC over a serial bus. The hand was fixed to a (unmoving) anthropomorphic robot arm by means of a three-axis force sensor (SOGEMI Srl, Italy), able to measure the forces exchanged between the hand and a human partner at the wrist level. The hand was placed 100 cm from the ground, in a way it could offer an object to a human partner (Fig. 3).

The data from the sensors were recorded (rate 1 kHz) by means of the data acquisition board (NI-USB 6211, National Instruments) and used by a custom PC application to control the release of the robotic hand during handover trials. The test-object was the same used in Experiment 1.

The robotic hand was programmed to automatically release the object following a stereotypical grip force trajectory. The release was triggered when the modulus of the force measured by the wrist sensor (*F*_w_) exceeded a certain threshold (*F*_wT_). At this time (*t*_release_) the robotic hand began decreasing the GF following a third-order polynomial trajectory (Fig. 3), akin to that observed in human-to-human handovers (Endo et al. 2012) (also confirmed by Experiment 1):

$$\begin{aligned} & {\text{GF}} ={a_0}+{a_1}{t^2}+{a_2}{t^3} \\ & {a_0} ={\text{G}}{{\text{F}}_0}, \\ & {a_1} = - \;\frac{{3{\text{G}}{{\text{F}}_0}}}{{T_{{\text{r}}}^{2}}}, \\ & {a_2} =\frac{{2{\text{G}}{{\text{F}}_0}}}{{{\text{T}}_{{\text{r}}}^{3}}}, \\ \end{aligned}$$

with *T*_r_, the release duration i.e. the time required to decrease the GF to zero (occurring at *t*_end_ = *t*_release_ + *T*_r_, defined as the duration of the handover). As both *F*_wT_ and *T*_r_ were fully programmable the robotic hand could implement different release behaviours by modulating two independent features: reactivity and speed. Indeed by changing *F*_wT_ it was possible to adjust *t*_release_, hence the beginning of the release, or in other words, the reactivity of the robot hand. In addition modulating *T*_r_ meant obtaining slower or faster releases.

#### Experimental protocol

In this test the robot hand played the role of the passer, while the participants of the receivers, in robot–human handovers. The participants, standing in front of the experimental materials on a marked position, were instructed to reach out, grasp and collect the test-object from the robotic hand, using their own dominant arm. In particular, for each experimental trial, the object was securely fixed in the hand (by the experimenter) using a pinch grip (GF_0_ = 6.5 N). Participants were instructed to execute the task at a self-paced speed and as naturally as possible.

The protocol included 12 conditions (C-*t*1,…,C-*T*4), randomized across participants, which differed based on the reactivity (*t*_release_) and speed (*T*_r_) exhibited by the robotic hand (Table 2). Each condition included two consecutive series (S1 and S2), of 2 and 10 trials, respectively, for a total of 144 trials. After each series an evaluation questionnaire was completed (for a total of 24 questionnaires). The questionnaire after S1 aimed to assess the fluency of the handover experienced by the receiver to a semi-novel yet poorly predictable passer’s behaviour (Huber et al. 2008b, 2013; Glasauer et al. 2010). The questionnaire after S2 instead, assessed the perceived fluency for stereotyped and predictable handovers.

**Table 2:** Handover conditions

Condition	*F*_wT_ (N)	Handover reactivity (*t*_release_ (ms) mean ± standard error)	Release duration *T*_r_ (ms)	Handover duration [*t*_end_ (ms)]
C-*t*1	0.8	74 ± 15	155	229
C-*t*2	0.8	74 ± 15	355	429
C-*t*3	0.8	74 ± 15	555	629
C-*t*4	0.8	74 ± 15	755	829
C-*Tt*1	0.34	29 ± 5	200	229
C-*Tt*2	0.34	29 ± 5	400	429
C-*Tt*3	0.34	29 ± 5	600	629
C-*Tt*4	0.34	29 ± 5	800	829
C-*T*1	0.8	74 ± 15	200	274
C-*T*2	0.8	74 ± 15	400	474
C-*T*3	0.8	74 ± 15	600	674
C-*T*4	0.8	74 ± 15	800	874

The questionnaire included the three following statements:

- Q1: “I am satisfied with the handover”;
- Q2: “The handover took place in a natural way”;
- Q3: “I perceived the actions of the robot to be…”

For Q1 and Q2 the participants were asked to rate the extent to which the statements did or did not apply, using a nine-point analogue scale. On this scale, 1 meant “absolutely certain that it did not apply”, 5 meant “uncertain whether it applied or not”, and 9 meant “absolutely certain that it applied”. Q3 had three possible answers:

1. “…early with respect to my action”;
2. “…coordinated with my action”;
3. “…delayed with respect to my action”.

To test release behaviours characterized by different reactivity and different release or handover duration, the 12 conditions mixed two different values of *F*_wT_ (0.34 N; 0.8 N) with several values of *T*_r_ and, consequently, with several values of *t*_end_. In particular, each value of *F*_wt_ was paired both with four values of *t*_end_ (229, 429, 629, 829 ms; C-*t*1,…,C-*t*4, C-*Tt*1,…,C-*Tt*4 in Table 2) and with four values of T_r_ (200, 400, 600, 800 ms, C-*Tt*1,…,C-*Tt*4, C-*T*1,…,C-*T*4 in Table 2). The tested *F*_wT_ values were chosen after a pilot study and corresponded to *t*_release_ values of 29 ± 5 ms (for *F*_wT_ = 0.34 N) or 74 ± 15 ms (for *F*_wT_ = 0.8 N). The *T*_r_ values were chosen based on the GF release duration observed in healthy humans, which is known to range between ~ 200 and 400 ms (Chan et al. 2012, 2013; Endo et al. 2012). Nonetheless we also tested longer *T*_r_.

#### Data analysis

##### Statistical analysis

The ratings on the perceived quality and timing of the handover were analysed using two three-way repeated measures ANOVAs. With the first ANOVA (ANOVA1), performed on controllers C-*t*1,…,C-*Tt*4, we tested the influence of *t*_release_, *t*_end_, and of the series. With the second ANOVA (ANOVA2), performed on controllers C-*Tt*1,…,C-*T*4, we tested the influence of *t*_release_, *T*_r_, and of the series. When the data violated the sphericity assumption, the Greenhouse–Geisser correction was used to adjust the degrees of freedom of the test. Moreover, the simple main effects of *t*_release_ at each level of *T*_r_ or *t*_end_ were tested applying the Bonferroni correction. Statistical significance was defined for *p* value < 0.05.

## Results

### Experiment 1

**Fig. 4:**
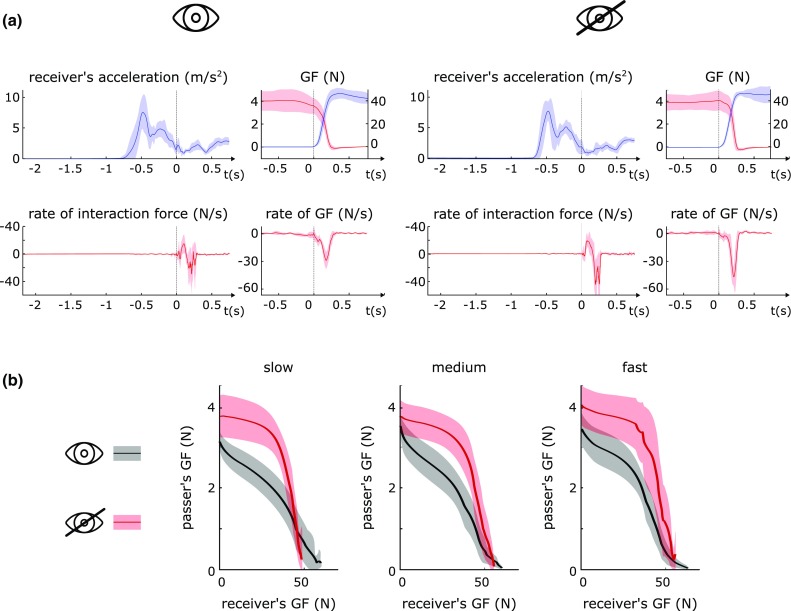
Representative outcomes from a single subject. **a** Representative profiles of receiver’s acceleration, passer’s and receiver’s grip force (GF), rate of passer’s GF and rate of interaction force during the fast receiver’s reach, under VT and *T* conditions. Bold line represents the mean; shadowed area represents the standard deviation. **b** Handover coordination profiles: passer’s vs. receiver’s GFs. The shadowed area represents the 95% confidence interval

Passers successfully completed handovers initiated by the receiver using slow, medium or fast reaches, both with and without visual sensory input. Illustrative functions in Fig. 4 show passer and receiver GF functions were consistently coordinated across receiver reach speeds, with passer reduction in GF starting earlier and progressing more steadily with vision. Without vision, the passer’s GF decrease was slower in onset, and progressed initially at a slower, then later at a more rapid rate.

**Fig. 5:**
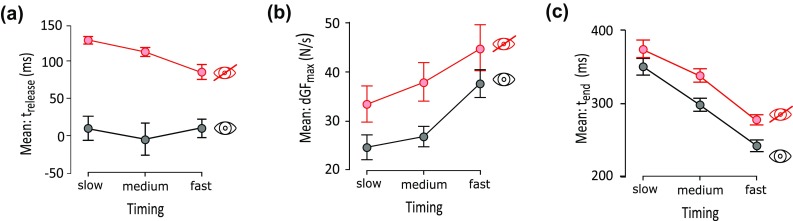
Effect of the visual condition and of the handover timing on the *t*_release_ (**a**), *t*_end_ (**c**), dGF_max_ (**d**) and *T*_t_ (**d**) and interaction between visual condition and group on *t*_release_ (**b**) (mean ± SEM)

The passer’s reactivity (*t*_release_) was affected by both the sensory input (repeated ANOVA, *F*(1,13) = 61.307, *p* < 10^−5^) and by the reaching out speed (repeated ANOVA, *F*(2,26) = 5.338, *p* = 0.011) (Fig. 5a) and the interaction between the two factors was significant (repeated ANOVA, *F*(2,26) = 3.699, *p* = 0.039). As expected, the post-hoc tests revealed higher reactivity when vision was available (*p* values < 10^−4^). Interestingly, the *t*_release_ did not differ across the three reaching out speeds (*p* values > 0.6), exhibiting an average value of 4.6 ± 15.6 ms (mean ± SEM). When participants were blindfolded the average reactivity was 108 ± 5 ms and increased with the speed of the movement; however, a significantly lower *t*_release_ was found only between the slow and fast movements (Fig. 5a). The *p* values of the post-hoc comparisons are listed in Table 3.

**Table 3:** Bonferroni corrected *p* values of post hoc multiple comparisons

Post hoc comparisons									
	VT + slow VT + medium	VT + slow VT + fast	VT + medium VT + fast	*T* + slow *T* + medium	*T* + slow *T* + fast	*T* + medium *T* + fast	VT + slow *T* + slow	VT + medium *T* + medium	VT + fast *T* + fast
*t*_release_	0.642	0.989	0.981	0.207	0.003*	0.066	0.000*	0.000*	0.000*
dGF_max_	0.213	0.000*	0.000*	0.012*	0.012*	0.084	0.018	0.003*	0.097
*t*_end_	0.000*	0.000*	0.000*	0.003*	0.000*	0.000*	0.116	0.002*	0.002*

*Statistically significant comparison (i.e. *p* < 0.05)

The kinetics of the handover (dGF_max_) was affected by the sensory input (repeated ANOVA, *F*(1,13) = 7.985, *p* = 0.014) and the reaching out speed (repeated ANOVA, *F*(2,26) = 30.026; *p* < 10^−6^), however, there was no interaction between the two factors (Fig. 5b). Although the dGF_max_ increased with speed for both the sensory input conditions, this was statistically significant in all comparisons with the exception of those between medium and fast under condition *T* (*p* = 0.08) and between slow and medium under condition VT (*p* = 0.2) (Fig. 5b). Interestingly, participants released their hold on the object faster when blindfolded; this was statistically true when the receiver’s reaching out movement was slow or medium (*p* values < 0.019) (Fig. 5b).

As for *t*_release_, the handover duration (*t*_end_) was affected by the sensory input (repeated ANOVA, *F*(1,13) = 10.701, i = 0.006) and the reaching out speed (repeated ANOVA, *F*(2,26) = 87.124, *p* < 10^−11^) with no significant interaction (Fig. 5c). The post hoc tests revealed that *t*_end_ decreased as the receiver moved faster for both the sensory input conditions (*p* values < 0.004); *t*_end_ was statistically higher in *T* than in VT for medium and fast movements (Table 3; Fig. 5c).

**Fig. 6:**
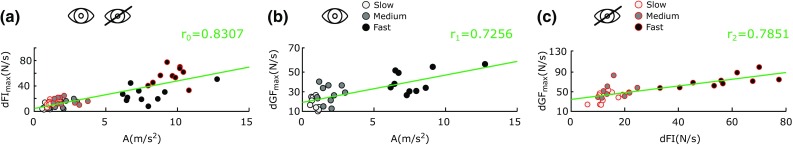
Representative linear correlation between dFI_max_ and *A* (*r*_0_), between dGF_max_ and *A* (*r*_1_) in condition VT and between dGF_max_ and dFI_max_ (*r*_2_) in condition *T* for one subject

Figure 6 shows illustrative scatter plots of the relations between (a) the peak interaction force rate and receiver’s peak reach acceleration (across sensory conditions), (b) the passer’s peak GF rate and receiver’s reach acceleration and (c) the receiver’s GF rate and the interaction force. Each of the three correlations is significantly greater than zero. The average correlation coefficient between the receiver’s reaching out acceleration and the magnitude of the tactile/haptic input, *r*_0_, proved significant (*r*_0_^m^ = 0.6549, *p* < 0.0001) (Fig. 6).

The average correlation (*r*_1_) between the receiver’s acceleration and the passer’s releasing speed, under the VT condition, proved statistically significant (*r*_1_^m^ = 0.584, *p* < 10^−3^), thus strengthening the outcomes of the statistics on dGF_max_ (Fig. 5b). The average correlation (*r*_2_) between the magnitude of the collision and the passer’s releasing speed, under the *T* condition, proved statistically significant (*r*_2_^m^ = 0.444, *p* = 0.014), again, in agreement with the previous test (Fig. 5a).

### Experiment 2

**Fig. 7:**
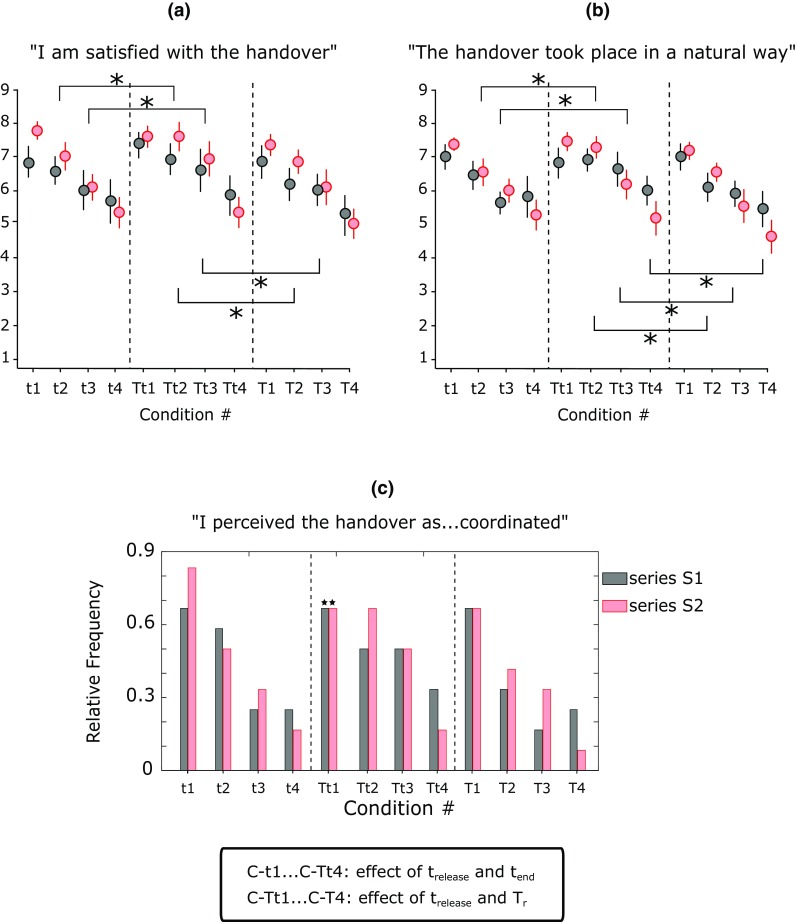
Subjective ratings of handovers with robot conditions 1–4 varying in duration from short (fast) to long (slow), and conditions *t* and *T* involving high tangential trigger force for onset of GF release (low reactivity) and *Tt* involving low trigger force (high reactivity) (mean ± SEM) for Q1 (**a**), Q2 (**b**), and relative frequencies of ratings for Q3 (**c**). Answers to block B1 in black, block B2 in red. Asterisk indicates statistically significant comparisons (corrected *p* values < 0.05); star indicates the series when at least one subject replied “early” to question Q3

For each condition and after each series, the ratings to questions Q1 and Q2 proved similar (Fig. 7). Though very short handovers were not judged as not coordinated (or unnatural), whereas long ones were so. Ratings of satisfaction, naturalness and perceived degree of coordination increased with: (1) more reactive release behaviours, (2) shorter release durations, and with (3) shorter handover durations (Fig. 7a, b).

In particular, the ratings of both Q1 and Q2 were statistically affected by *t*_release_ (ANOVA1, *F*(1,10)-values > 9.82, *p* values < 0.011; ANOVA2, *F*(1,10)-values > 12.19, *p* values < 0.01), *t*_end_ (ANOVA1 for Q1, *F*(3,30)-values > 4.889, *p* values < 0.01; ANOVA2 corrected for Q2, *F*(1.702,17.025) = 13.129, *p* < 0.001) and *T*_r_ (ANOVA2 corrected for Q1 *F*(1.74,17.4) = 24.455, *p* < 0.001; corrected for Q2, *F*(1.603,16.028) = 15.918, *p* < 0.001). For question Q1, when comparing conditions with same handover duration (*t*_end_) but different reactivity (*t*_release_) we found a significant preference for more reactive releases in those handovers with *t*_end_ equal to 429 ms and 629 ms (*p* values < 0.05). For the fastest (*t*_end_ = 229 ms) and the slowest (*t*_end_ = 829 ms) conditions it made no difference whether the handover was more or less reactive. Equivalent results were found when comparing conditions with same release duration (*T*_r_) but different *t*_release_ (Fig. 7a). Matched results were also found for Q2 (Fig. 7b).

The statistical analysis did not reveal significant main effects of the series (*F*(1,10)-values < 2.93, *p* values > 0.7) on neither ratings of Q1 and Q2. However, for both Q1 and Q2 a significant interaction was found between *T*_r_ and the series (ANOVA2 *F*(3,30)-values > 3.823, *p* values < 0.02) and between *t*_end_ and series (ANOVA1 *F*(3,30)-values > 3.155, *p* values < 0.04). In fact, for faster release behaviours participants provided larger ratings after S2 than after S1; for slower controllers, ratings proved higher after S1 than S2 (Fig. 7a, b).

The qualitative analysis of the answers to Q3 supported the outcomes of the statistical tests for Q1 and Q2. In particular, the number of participants who perceived the robot as “coordinated” decreased with: (1) decreased reactivity, (2) increased release time, and (3) increased completion time of the handover (Fig. 7c). Remarkably only two participants in S1 and one participant in S2 reported that fastest condition was early with respect to their actions.

## Discussion

In this work, we investigated the passer’s GF modulation with varying receiver’s reaching out speeds and different sensory input conditions, as well as the receiver’s perception of the fluency of the handover with different releasing behaviours. In the first experiment, we investigated whether the passer integrates the visual input about the receiver’s approaching movement to select (in advance) the release motor program, and compared the GF profiles with those of a blindfolded passer.

### Release behaviour with visual and tactile input

Taken together our results are compatible with the control models for manipulative tasks (Johansson and Edin 1993; Randall Flanagan et al. 2006; Johansson and Flanagan 2009; Fang et al. 2015; Haruno et al. 2001; Flanagan and Wing 1997). In the first experiment, we observed that passers endowed with both visual and haptic/tactile input use predictive/anticipatory control purely based on vision to trigger the handover (Figs. 4b, 5c). This outcome extends Mason’s and MacKenzie’s (2005) study because it demonstrates that when vision is available the passer estimates and synchronizes the release of the object with the time of the collision, independently of the receiver’s reaching out speed (Fig. 5a). Such a predictive behaviour shows similarities with the one observed during catching tasks at different speeds, hence similar mechanisms may explain it (Lacquaniti and Maioli 1989; Savelsbergh et al. 1992). For example Savelsbergh et al. (1992) suggested that the prediction of the collision is based on the time-to-contact and not on the distance-to-contact. The fact that the passer does not wait for the collision to begin the handover, implies that online tactile/haptic input is not used by the nervous system to select and launch the appropriate release motor program. Instead, such an input may be used only for monitoring the execution of the motor task and, if necessary, to initiate corrective actions (Johansson and Edin 1993; Randall Flanagan et al. 2006; Johansson and Flanagan 2009; Fang et al. 2015). This hypothesis is in agreement with the study of Endo et al. (2012), who showed that a partial corruption of the passer’s tactile/haptic input does not affect the beginning of the release albeit it increases the passer’s uncertainty at the end of the handover. However, it remains to be clarified how passers would change the way they release if they performed handovers in total absence of tactile input.

Our results show that in normal visual conditions a passer scales the releasing speed (dGF_max_) according to the acceleration of the receiver’s reaching out movement (Figs. 5b, 6). This suggests that while the receiver reaches for the object the passer, observing the receiver’s movement, infers the dynamics of the upcoming handover and thus selects the appropriate releasing speed. This hypothesis is in agreement with the theory of Quesque and his group (Quesque and Coello 2015; Quesque et al. 2016) that one’s intention can be anticipated from the observation of the partner’s action. In addition it is consistent with the kinematical correlations found between cooperative agents (Georgiou et al. 2007) and with the hypothesis of *motor resonance*. The latter posits that when an individual observes other people’s movements, his/her brain runs an internal motor simulation, used to understand the other’s people intentions and to predict the future course of the observed action (Rizzolatti and Craighero 2004; Springer et al. 2012).

In their work, Mason and MacKenzie studied human-to-human handovers under normal visual conditions, identifying them as coordinated by demonstrating synchronized peaks of the GF rates. Here we suggested a more illustrative way to describe the coordination between the agents, which is the passer’s GF expressed as a function of the receiver’s GF (the graphs in Fig. 4b). This methodology borrows from the neuroscience literature, which presents similar metrics (the GF vs. the load force) to assess motor coordination during grasp (Flanagan et al. 1993) and to compare, e.g. healthy vs. impaired participants, adults vs. children, unimpaired digits vs. anesthetized ones (Gordon et al. 2007; Cipriani et al. 2014). The coordination profiles observed under visual input confirm and once again extend the finding of Mason and MacKenzie, by showing that once collision has occurred the passer and the receiver modulate their GF in a synchronized manner, until the end of the handover, and regardless of the receiver’s reaching out speed (Fig. 4b). Again, this could be explained by the motor resonance hypothesis. Nonetheless, it seems that such a coordination slightly decreased for the fast reaching out movement, as demonstrated by less correlated GFs (the curves in Fig. 4b become less linear with speed). This invites further studies in which faster handovers are tested, to assess whether the motor task becomes less coordinated.

### Release behaviour with only tactile input

When only tactile feedback was available, the passers released the object exhibiting a sensory elicited (feedback-based) behaviour. The correlation found between the receiver’s reaching out acceleration and the magnitude of the tactile/haptic suggests that the information associated to the receiver’s movement may be transferred to the passer visually and/or via tactile/haptic interaction (Fig. 6). However, the tactile feedback was not enough for achieving coordinated movements, as clearly depicted by the coordination profiles (cf. the knees in the red curves in Fig. 4b). The delays observed when the passer was blindfolded, fell within the range of the latencies associated to automatically initiated responses elicited by somatosensory input during grasp (Johansson and Westling 1984, 1987, 1988a, b).

We also observed that the latency between the collision and the beginning of the release decreased with the speed of the receiver’s reaching out movement (Fig. 5a), and in turn with the magnitude of the collision (Fig. 6). Opposite trends were observed for the grip force rate (dGF_max_) (Fig. 5b) which scaled with the magnitude of the collision (dFI_max_) (Fig. 6). Interestingly, this release behaviour shows similarities with the *impulsive catch-up response*. The latter is a rapid increase of GF that adult humans adopt to stabilize their grasp on an object, when unpredictable load force perturbations occur (Cole and Abbs 1988; Cole and Johansson 1993). In particular, Johansson et al. (1992) showed that the latency between the start of the perturbation and the onset of the catch-up response decreases with the rate of the perturbation (akin to Fig. 5a), and that the peak rate of the GF is proportional to the magnitude of the perturbation (akin to Fig. 6). The complementarity in the outcomes may be explained considering that the goal of the catch-up test (i.e. to stabilize the hold after an unexpected perturbation) is opposite to the goal of the blindfolded passer (i.e. to release the hold after an unexpected perturbation). These results suggest that when visual input is unavailable, the passer’s neural system may involve fast feedback mechanisms akin to those involved in the catch-up response, to modulate the GF. Moreover, as already hypothesised by Johansson for explaining the catch-up response (Johansson et al. 1992), the GF response in the blindfolded passer may be elicited only if the sensory input associated to the collision exceeds a certain threshold. Thus, when the receiver produces a more intense collision, for example due to a fast reaching out movement, tactile/haptic signals overcome sooner such a threshold and the passer begins the release earlier (Fig. 5a).

### Perception of a fluent handover

In the second experiment, we investigated how the fluency of the handover perceived by the receiver reaching out at a self-paced speed is influenced by the reactivity, the speed of the release and the completion time of the handover. Results showed that control strategies with a lower *t*_release_ (i.e. a lower *F*_wT_ to trigger the release) were generally preferred.

The statistical analysis proved that the effect of *t*_release_ on the ratings was significant only when the handover was executed with the two intermediate levels of *T*_r_ and *t*_end_ (C-*t*2, C-*t*3, C-*tT*2, C-*tT*3, C-*T*2, C-*T*3) but not for the fastest and slowest conditions. This outcome advocates two discussion points. The first one is that the initial phase of the handover (immediately after the collision), influences the receiver’s perception of fluency only when the interaction takes place at a natural pace, and this was expected since it is coherent with the reaching out speed of the participants. When a latency is present, even very small, this is perceived and cognitively processed by the receiver, and the interaction is experienced as less natural.

The second interesting point refers to the observed lack of sensibility to reaction time in the slowest and fastest conditions. This suggests (but is just an hypothesis) that we probably found the range of perceivable differences in the stimuli (with the experienced fluency being the stimulus) achievable with the present parameters. For the slowest conditions, this may be due to the time scales involved: the subtle difference in reaction times (roughly 40 ms) was one order of magnitude lower than the handover duration (*t*_end_ > 800 ms); it is likely that for so slow handovers, the brain misses the saliency of slightly shorter or longer reaction times. For the fastest conditions instead, in view of the outcomes of Experiment 1, we argue that better ratings could be achieved for reaction times closer to zero. Indeed as observed in the first experiment, in normal conditions the reaction time is close to zero (predictive behaviour) for a wide range of handover dynamics. Hence handovers may be experienced as natural only when the passer uses a predictive behaviour and starts releasing the object at the time of the collision. With this in mind is was not surprising to learn that the experimental conditions were experienced as too fast only in three instances during the experiment. An additional reason may reside in the influence that an interaction with a robotic partner may have on the behaviour of the participants. Contrary to human–human handovers, in robot–human handovers it is likely that participants took the responsibility of the object stability with the result that they exploited a faster dynamics of the grasping force to mitigate the risk of dropping the object (that may occur if the robotic passer releases the object too soon). Thus, the release of the object occurred when the grasping force of the receiver was sufficient to stably hold the object, and fastest releases were judged as satisfactory and coordinated. In any case, the need of a predictive behaviour in a robot passer poses an important requirement to the designers of collaborative robots aimed to interact safely and naturally with humans. Vision or other sources of artificial sensory inputs may be used for achieving such a challenging goal.

The interaction found between the release duration (*T*_r_) and the level of confidence with the passer’s behaviour (i.e. series S1 and S2) suggests a way to split the conditions between those that were deemed acceptable and those that were not. The acceptable conditions were probably those for which participants were prone to adapt and synchronize with the robot hand behaviour. Hence these conditions achieved a greater score after ten repetitions (the second series) than after the very first two trials (S1). Vice versa the unacceptable conditions were those that got a worse mark after the second series.

Further researches are necessary to investigate the fluency of the handover perceived by the receivers when they have to perform also a subsequent task with the object. In particular, it would be interesting to determine the effects of such a perception on the kinematic of the receiver not only during the handover but also during the following actions.
